# Integrating community efforts, innovative government interventions and data in the fight against kala-azar: lessons from Dumka, India

**DOI:** 10.1093/inthealth/ihaf083

**Published:** 2025-09-24

**Authors:** Neha Arora, Ravi Shankar Shukla, Dhruv Pandey, Abhishek Paul

**Affiliations:** Indian Administrative Service, Additional Secretary, Drinking Water and Sanitation Department, Mission Director, SBM (G) and Additional Chief Election Officer, Government of Jharkhand, Ranchi, Jharkhand, India 834002; Indian Administrative Service, ex-Mission Director, National Health Mission, and ex-Deputy Commissioner, Dumka (present Deputy Commissioner Saraikella-Kharsawan) Government of Jharkhand, Saraikella-Kharsawan, Jharkhand, India 833219; Medical Officer, Tropical and Vector Borne Diseases, WHO ARFO, Pretoria, South Africa 0076; State Coordinator Neglected Tropical Diseases, WHO India, Ranchi, Jharkhand, India 834010

**Keywords:** Dumka, elimination, innovative interventions, kala-azar

## Abstract

Kala-azar, a sandfly-borne neglected tropical disease, primarily affects the poorest populations in endemic regions of India. In 2023, India achieved the elimination threshold as a public health problem by reporting <1 case per 10 000 population at the block level across the country. Dumka, one of four kala-azar endemic districts in Jharkhand, was a significant focus of transmission and achieved the target 1 y before, in 2022, sustaining it through today. Strategic, data-driven innovative interventions addressed persistent sociocultural and socio-economic factors that were contributing to the persistence of transmission in Dumka.

## Introduction

Kala-azar, also known as visceral leishmaniasis (VL), is a sandfly-borne neglected tropical disease that predominantly affects the most impoverished sections of India. Leishmaniasis manifests in India in two major forms: VL or kala-azar, and post-kala-azar dermal leishmaniasis (PKDL). PKDL occurs in 10–15% of kala-azar cases, serving as a reservoir for disease transmission.^1^

**Figure 1.**
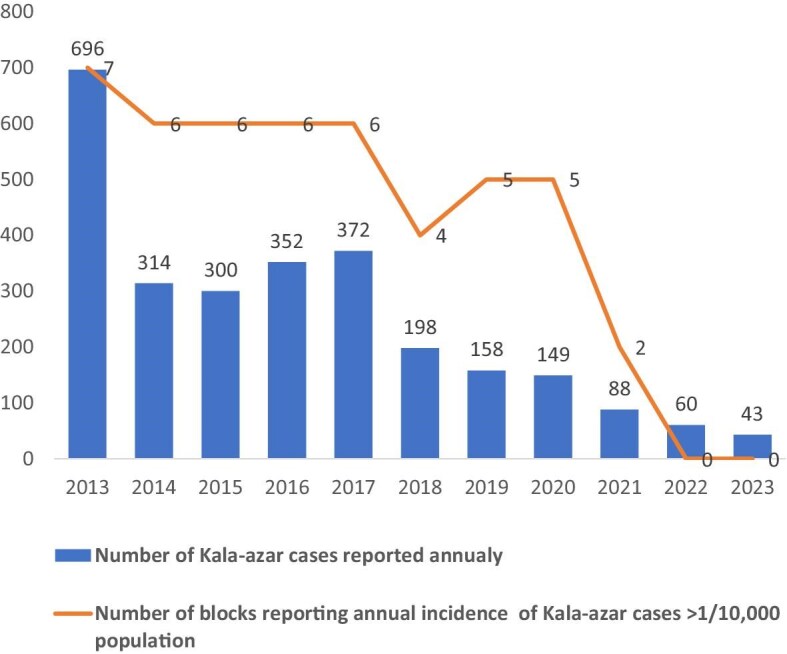
Number of kala-azar cases reported annually and number of blocks reporting an annual incidence >1 per 10 000 population from 2013 to 2023 in Dumka.

Significant progress has been made in kala-azar elimination in India due to the introduction of new interventions such as liposomal amphotericin B in 2014, inclusion of synthetic pyrethroid as an insecticide and hand compression pumps for indoor spraying in 2015–2016. The state governments of Jharkhand and Bihar and the central government introduced wage loss compensation for kala-azar patients treated free of cost under the government healthcare services umbrella. All these efforts have significantly contributed to the huge decline of kala-azar cases from 2515 in 2013 to 160 in 2023 in Jharkhand (a 93.6% decline) (Figure 1).^2^ According to the 2002 National Health Policy, kala-azar was targeted for elimination as a public health problem in 2010 and the deadline was revised a number of times, with latest being 2023.^3^ Elimination as a public health problem is defined as an annual incidence of <1 case per 10 000 population at the block level (subdistrict implementation unit for kala-azar elimination). In 2021, only 7 of 633 endemic blocks in the country reported an annual incidence above the elimination threshold and among these 7 blocks, 2 were in Dumka (i.e. Jama and Kathikund blocks reported an annual incidence of 1.46 and 1.33 per 10 000 population, respectively) (Figure 1).^2^

Dumka, one of four kala-azar-endemic districts in Jharkhand, has a predominantly rural population with a significant tribal community. Their living conditions—poorly ventilated homes and cattle sheds within the house—contribute to sandfly vector breeding. Healthcare-seeking behaviour favouring the informal sector presents challenges in early detection, treatment and transmission interruption in many communities.

Last-mile challenges in disease elimination are often seen as significant hurdles, particularly when diseases localize in certain areas, risking transmission spillover into already cleared regions. To address this, intensified and innovative strategies are essential for meaningful impact. In the Dumka district, after consultations with stakeholders, the deputy commissioner recognized that the health team alone could not achieve elimination without administrative and social support to integrate services and government schemes with cultural practices. Data-driven gap analysis facilitated the formulation of targeted strategies. As a result, there was a remarkable 93.8% decline in kala-azar cases from 2013 to 2023, with all blocks achieving elimination 1 y ahead of schedule. This article highlights the effectiveness of data-driven targeted interventions and the importance of integrating various services to overcome last-mile challenges and sustain kala-azar elimination.

## Strategic interventions

The Dumka district implemented a comprehensive strategy to reduce kala-azar transmission by enhancing current interventions under the National Kala-azar Elimination Program and introducing innovative, sustainable approaches. These efforts aligned with the Accelerated Kala-azar Elimination Plan 2017 and the World Health Organization's (WHO) regional framework for elimination.^5^

### Strengthening the surveillance system to ensure early case detection and prompt treatment

Analysis of kala-azar cases reported from Dumka revealed that >70% were from tribal and primitive groups.^2^ To improve health-seeking behaviours and facilitate timely diagnoses, the administration engaged informal health sectors through regular sensitization and follow-ups. Approximately 250 rural health providers were oriented, while formal sectors received training through workshops involving the Indian Medical Association and Jharkhand State Health Services Association to enhance case reporting. Active case detection is vital for early identification and reducing transmission. The administration conducted six house-to-house search campaigns guided by the National Centre for Vector Borne Disease Control. Field teams, comprising two members each, were tasked with symptom inquiries and documenting findings, supervised at various levels. Mobile medical units provided diagnostic support, while medical officers wore identifiable attire to boost community confidence. In Dumka's hilly terrain, health camps replaced traditional house-to-house methods. A cash incentive of 1000 Indian rupees (about US$13) was introduced for suspect referrals and awards for confirmed cases expedited identification. Field teams used the online Kala-azar Management Information System (KAMIS) for real-time monitoring.

### Addressing challenges of indoor residual spraying (IRS)

The administration enhanced IRS by reducing the coverage to 15 houses per pump per day from the usual 20. This change improved spraying quality, addressing issues related to challenging terrains. To increase community acceptance, pre-information stickers providing advance notice were created, highlighting the benefits of the IRS, and placed on doors 2 d before spraying. Operational challenges leading to incomplete sprayings, such as locked houses and refusals, were tackled by introducing a ‘mobile spray team’ to convert partially sprayed houses into fully treated ones. Entomological surveillance was improved with the support of the state health team to ensure both pre- and post-IRS assessments.

### Streamlining of case management of kala-azar and PKDL and addressing relapse cases

Kala-azar is a rare disease in non-endemic regions, and most medical officers have limited experience in managing kala-azar and PKDL, particularly in patients with comorbidities like human immunodeficiency virus, tuberculosis and diabetes. To address this, orientation workshops and training sessions were conducted with support from Indian Council of Medical Research (ICMR) institutes and WHO teams. Kala-azar patients often require blood transfusions due to moderate to severe anaemia, and special provisions were made for issuing blood units despite donor unavailability. Nutritional kits containing locally available cereals and millets were distributed to improve the health of treated patients, considering the high prevalence of malnutrition and alcoholism in affected communities. PKDL cases, which usually present mild and cosmetic symptoms, were emphasized in surveillance to ensure early detection, as 5–10% of treated kala-azar cases may develop PKDL within 2–3 y. Relapse cases, defined by a recurrence of symptoms after treatment, pose a risk of infection, and similar treatment dosages are recommended for new and relapse cases.^1^ A kala-azar expert committee was also established to oversee treatment for difficult cases and those with comorbidities.

### Building a sustainable and resilient environment

A holistic approach was needed to address the sociocultural and socio-economic factors contributing to sandfly breeding. A socio-economic survey in 13 key villages revealed that >87% of kala-azar cases in 2021 and 2022 lived in mud or mixed houses, which favour vector breeding. In response, the district administration initiated construction of concrete houses for patients through the ‘Ambedkar Awas Yojana’ and allocated 66 houses under the Pradhan Mantri Awas Yojana Gramin (PMAY-G) scheme. Additionally, insecticide-treated bed nets were distributed and Open Defecation Free (ODF) Plus initiatives improved sanitation and waste management, further reducing vector breeding. Measures were also taken to tackle multidimensional poverty by constructing toilets, soak pits and cattle sheds.

### Social mobilization

Village-level governing bodies, including Panchayati Raj Institution members, local self-help groups under the Jharkhand State Livelihood Promotion Society, Accredited Social Health Activist volunteers and Anganwadi workers, were engaged in community mobilization and awareness for kala-azar elimination. Kala-azar awareness mobile melas were held to highlight the disease's seriousness, the benefits of spraying and the importance of community. Monthly village meetings focused on kala-azar discussions and a yearly ‘Marathon Run for Kala-azar free Dumka’ involved civil societies, non-governmental organizations, media and affected communities to raise awareness. Regular radio messages, along with audio and video content, were created and shared in collaboration with the fertilizer cooperative to effectively and inclusively reach the agrarian community.

### Program review and monitoring

For real-time reporting and follow-up, the district administration used KAMIS^2^ and an information communication technology–based mobile app. A dedicated help-line was established for disease identification, treatment and prevention. Development partners monitored field activities and provided weekly reports. Field staff shared geo-tagged photos in social media groups. Monthly district task force meetings led by the deputy commissioner tracked progress and addressed gaps, with similar meetings at the block level for detailed oversight. Regular analysis by stakeholders offered valuable feedback for necessary adjustments.

## Conclusions

Interventions led to significant reductions in kala-azar transmission in the Dumka district (from 158 cases in 2019 to 43 in 2023; a 72.3% reduction).^2^ In 2019, five blocks had an annual incidence above the elimination threshold, whereas since 2022, none have reported an incidence above the threshold.^2^ The average duration of fever before diagnosis decreased from 36 d in 2019 to 30 d by 2023 and the average treatment delay (diagnosis to treatment initiation) has decreased from 5 d in 2019 to 2 d in 2023.^2^ A total of 103 PKDL cases were identified from 2021 to 2023, which is 92.8% of the total PKDL cases reported between 2018 and 2020 (116 cases).^2^

The elimination of kala-azar in India is guided by evidence-based practices aligned with WHO strategies. The administration has focused on data-driven interventions, regular reviews and community engagement. Targeted strategies, like contact tracing and providing adequate housing, were developed based on data analysis. In the short term, strengthening of the surveillance system and IRS produced the most significant impact (Table 1). Progress so far indicates that by intensifying strategic interventions, modifying ground-level practices and integrating approaches with strong coordination, we can overcome long-standing challenges in disease elimination. Changes in transmission trends show that our efforts are effective, and with continued commitment, the district should soon achieve ‘interruption of transmission’. The interventions can be replicated in all kala-azar-affected areas, especially in similar communities and among populations with poor health-seeking behaviour. The interventions were directed to bring sustainable changes by modifying the ecological conditions and addressing multidimensional poverty-related factors. However, long-term sustainability of interventions relies on consistent funding and support alongside improvements in entomological surveillance for early detection of potential outbreaks. Future research should focus on developing improved treatment regimens for both new and relapsed cases, as well as exploring the long-term socio-economic impacts of interventions such as housing improvements, which can help reduce sandfly habitats. Any complacency at this stage could jeopardize years of progress due to the disease's long incubation period and the community's vulnerability.

**Table 1:** Summary of strategic interventions implemented to reduce kala-azar transmission in Dumka.

Interventions	Short description	Outcomes
Strengthening the surveillance system	Including formal and informal healthcare providers according to local context; introduction of incentive schemes; deployment of mobile medical units; camp approach for hard-to-reach areas	Reduction in diagnosis and treatment delays; improved surveillance system sensitivity. Average fever duration before diagnosis decreased from 36 d in 2019 to 30 d by 2023. Average treatment delay decreased from 5 d in 2019 to 2 d in 2023
Addressing challenges of IRS	Reduced daily coverage target; use of pre-information stickers on the benefits of IRS; introduction of a ‘mobile spray team’ to convert partially sprayed houses; timely entomological surveillance	Enhanced quality and coverage of IRS and reductions in refusals by the community. Independent evaluated coverage reported >80% full surface coverage
Streamlining of case management	Conducting of orientation workshops and training sessions for individuals involved; special provisions for issuing blood units despite donor unavailability; provision of nutritional kits for treated patients; formation of an expert committee to oversee treatment of difficult cases and those with comorbidities	Improved diagnostic and management capabilities; improvement in post-treatment outcomes and prognosis
Building a sustainable and resilient environment	Socio-economic survey in 13 key villages to understand and estimate vector-favourable ecological conditions; concrete houses, soak pits and cattle sheds built under various government schemes; provision of insecticide-treated bed nets; ODF Plus initiatives	Modification of vector-favourable ecology in affected villages helped in reducing transmission. A total of 66 pucca houses for patients were built under the state scheme, i.e. Ambedkar Awas Yojna apart from PMAY-G allotment
Social mobilization	Village-level governing bodies with locals engaged in the community; kala-azar awareness mobile melas; yearly ‘Marathon Run for Kala-azar free Dumka’; regular radio messages	Improved health-seeking behaviour and community ownership
Program review and monitoring	Use of digital program management systems; dedicated helpline established for disease identification, treatment and prevention; regular program review using field visit reports of all stakeholders	Real-time course correction and improved efficiency in program management

## Data Availability

The data underlying this article are available in the article.
